# Entrainment is sparse

**DOI:** 10.3389/fnhum.2014.00618

**Published:** 2014-08-11

**Authors:** Eric Barnhill

**Affiliations:** ^1^Clinical Research Imaging Centre, Department of Clinical Sciences and Community Health, College of Medicine and Veterinary Medicine, The University of EdinburghEdinburgh, UK; ^2^Institute for Music in Human and Social Development, Reid School of Music, Edinburgh College of Art, The University of EdinburghEdinburgh, UK

**Keywords:** entrainment, joint sparse representation, neuromusic, music therapy, music, music cognition, neural coding

## 1. Introduction

Entrained behavior coordinates, predicts, and modulates multi-scale rhythmic gestures with high spatio-temporal precision even as it shows flexible adaptation in response to perturbation (Clayton et al., 2005; Altenmüller et al., 2006; Phillips-Silver et al., 2010). The capacity for this split-second, multi-scale timing is often viewed as a highly-complex, specialized virtuosity that emerged in the forges of natural selection for evolutionary advantage (Mithen, 2005; Knoblich and Sebanz, 2008; Merker et al., 2009). Entrainment has found compelling mathematical models in the interaction of multiple dynamic oscillators (Large, 2010) and convincing neurological substrata in the electrophysiological resonance patterns that support cognition (Nozaradan et al., 2011; Schaefer et al., 2011). Further, entrainment-based therapeutic interventions have been validated in both quantative (Thaut and Abiru, 2010) and qualitative (Aigen, 2008) studies.

This paper aims to bolster the theoretical case for the transformational potential of entrainment therapy by casting it in the framework of contemporary engineering mathematics, in particular applying the concepts of change of basis, Fourier transform, and most importantly, the growing body of work on Joint Sparse Representation (JSR) (Bruckstein et al., 2009). The paper aims to be a conceptual introduction in the hopes of reaching a wider audience that may want to make use of the relationship between entrainment and sparsity, and apply more engineering mathematics to their analyses of entrainment in therapy and performance.

## 2. Three key concepts

### 2.1. Change of basis

Many of the engineering marvels around us have, as a keystone of their mathematical foundations, a change of basis (Kreyszig, 2007). A technical definition of a mathematical basis is a set of linearly independent vectors within a space that, in combination, can span the entirety of that space. For example, the Cartesian basis for three-dimensional real space (aka *R*3) is a set of three orthogonal (perpendicular) unit (length of one) vectors, pointing along the x, y, and z axes respectively. In vector notation the orthonormal Cartesian *R*3 vectors are [1 0 0], [0 1 0] and [0 0 1]. We say these vectors *span R*3, as any point in *R*3, for which we have the coordinates [x y z], can be reached from the origin using the vectors [x 0 0] + [0 y 0] + [0 0 z]. The Cartesian *R*3 basis is, in other words, the way we might account for spatial activity using rulers or graph paper. Add a fourth dimension for time, to span spatio-temporal activity, and the same rules apply for any vector [x y z t].

### 2.2. The fourier basis and the frequency domain

This spatio-temporal Cartesian basis is our most intuitive approach to representing the world around us, but also a very poor representation for solving many engineering problems. One of the most commonly used changes of basis is the family of Fourier or frequency-domain transforms, in which a function is represented on a basis of sinusoidal *periodic functions* rather than units of Cartesian distance. In its discrete form, a signal is transformed from a series of consecutive sampled values into a combination of sinusoids of different amplitudes and frequencies.

**Figure 1:**
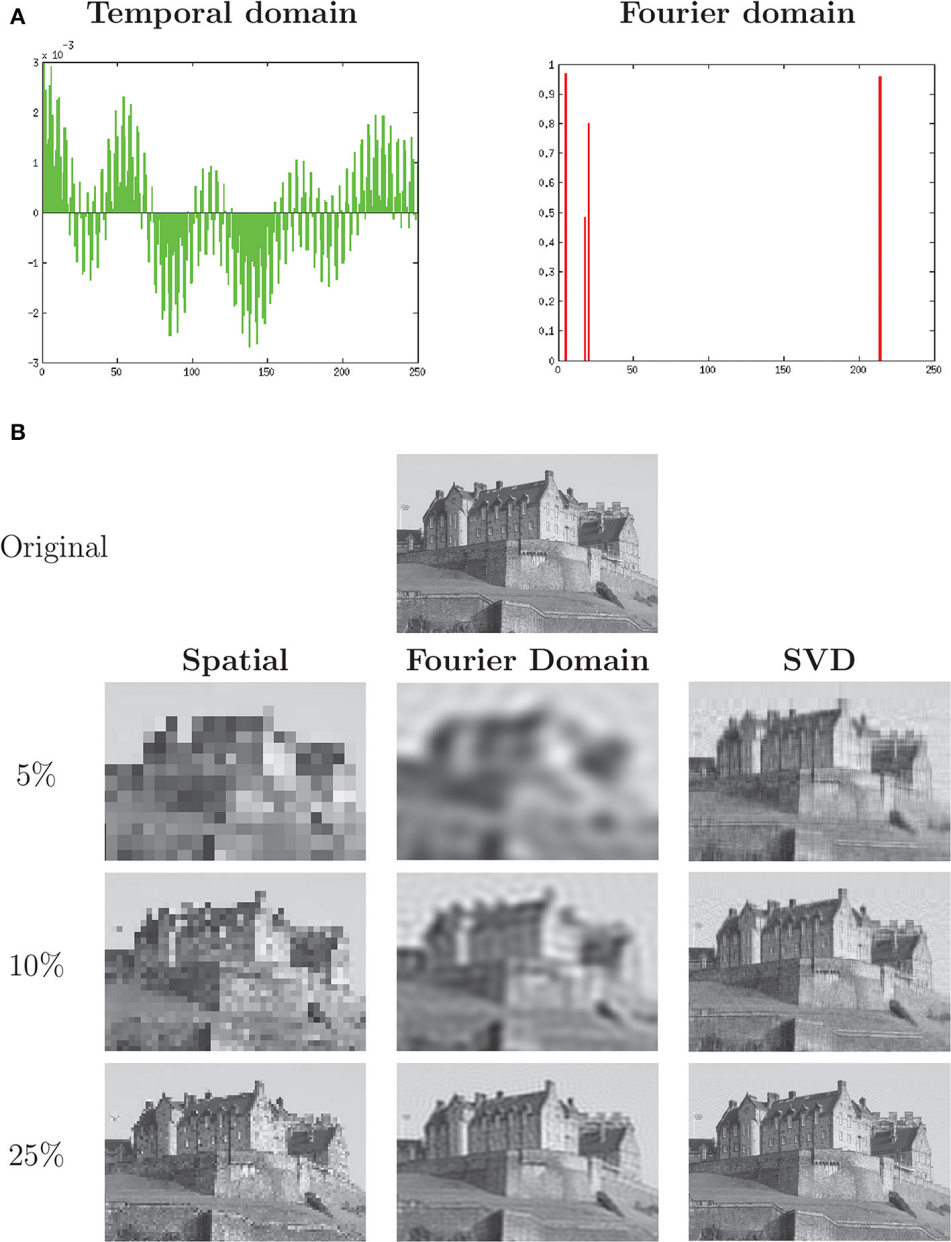
**(A)** A dense periodic discrete signal may have a sparse representation when transformed into the Fourier domain (DCT-II transform). **(B)** Image of Edinburgh Castle, with Spatial (Cartesian basis), Discrete Cosine Transform (frequency domain basis) and Singular Value Decomposition (least-squares optimal basis) compression applied at decreasing compression rates. Source: Stuart Caie, CC BY 2.0 license. Reproduced grayscale with described modifications.

While conceptually cumbersome at first, Fourier transformation has many advantages for not only the analysis, but the storing and compression, of many kids of data. Take, for example, a sample of a single musical note, vibrating at a particular frequency, that would appear on an oscilloscope as a complex periodic waveform. In the time domain, this signal will be dense, that is, it will contain few if any zeros and most of the signal will be required for its reconstruction as specified by the the Nyquist-Shannon sampling theorem (Shannon, 1949). If, however, the signal is like most signals coming from a musical instrument—a combination of a fundamental frequency and a small number of overtone frequencies—then it can be represented in the Fourier domain with a small number of values, one for each component frequency, leaving the rest of the basis vectors at zero magnitude. The signal vector thus meets the mathematical definition of *sparse*—most of its coefficients are zero—and its representation can be efficiently compressed, requiring far less data for its representation than the Nyquist theorem specifies. Figure 1A illustrates the relationship between a complex periodic waveform and its sparse Fourier transformation.

Mathematically, a signal and its Fourier transform are one-to-one mappings. The frequency-domain representation of the signal is often much more efficient, however, in the sense that far more of the signal information is packed into a small subset of the vectors that span the basis. JPEG (Skodras et al., 2001) and MPEG (Le Gall, 1991) compression schemes, for example, discard well over 90% of the information within a signal in part by transforming that image into the frequency domain (DCT in the case of MPEG and Daubechies wavelet in the case of JPEG 2000) and eliminating the many frequency bands of near-zero magnitude. The resulting compressed data formats still retain enough of the significant information to have become the *lingua franca* of images and music, respectively. An example of data compression in the spatial frequency domain is seen in Figure 1B.

Frequency-domain transform is hard-wired into the anatomy of the cochlea, whose hair cells of varying stiffness resonate with stimuli of specific frequencies, triggering action potentials via auditory transduction. The inner ear thus performs a frequency transform of incoming auditory information across a small temporal window, known in its simplest form as a short-time Fourier transform (STFT), though actual observed performance resembles a somewhat more complex transform known as time-frequency reassignment (Auger et al., 2013).

### 2.3. Sparse overcomplete coding

If sufficient information about the signal can be deduced from a small portion of a signal via a mathematical transform, then the benefits to the actor are obvious. Both computationally and metabolically, the organism that can reduce processing demands by such a large amount can expect to reap benefits. If frequency-domain and similar bases yield such improvements in information coding efficiency, the key question for modeling neural coding is to ask how that information might be coded to its optimum.

The optimal basis for a signal in a least-squares sense is its Singular Value Decomposition (SVD) (Strang, 2007). A comparison of spatio-temporal, frequency-domain and SVD data compression is shown in Figure 1B. The frequency-domain images show many more features at each level of compression than the Cartesian (nearest-neighbor) compression, while the SVD images show substantially more than either.

However, the SVD of a single signal is not necessarily the sparsest representation of that signal in the context of a set of signals such as that encoded in neural memory. Much greater compression can be attained through the re-use of common basis vectors to transform many signals. In this approach the process of neural memory is modeled as manipulation of a set of learned basis vectors known as a “dictionary,” in which incoming signals are decomposed in the sparsest possible way using the atomic vectors or “atoms” that make up the dictionary (Rubinstein et al., 2010). This operation is non-linear but many efficient algorithms have been developed for sparse dictionary coding, primarily through *L*1-norm minimization (Donoho and Elad, 2003). The most efficient dictionary systems are found to be “sparse overcomplete,” that is, they consist of many more basis vectors than necessary for the set of signals, but have great flexibility to maximize the sparsity with which an incoming signal is encoded (Bruckstein et al., 2009; Rubinstein et al., 2010).

Finally the atoms of the dictionary must adapt to the new signals in accordance with the principles of Hebbian and Bayesian learning. Efficient algorithms have been discovered for this process as well, whether the classic K-SVD (Aharon et al., 2006) or more recent parametric or multiscale dictionary updating algorithms (Rubinstein et al., 2010).

Perhaps unsurprisingly, there is abundant experimental evidence for such sparse coding in human and animal brains (Olshausen and Field, 2004). Evidence supporting a sparse coding model has been found in studies of visual (Olshausen and Field, 1997; Vinje and Gallant, 2000), auditory (Hromádka et al., 2008), olfactory (Ito et al., 2008; Poo and Isaacson, 2009), haptic (Jadhav et al., 2009; Crochet et al., 2011) and motor (Hahnloser et al., 2002) processing. Sparse coding models relate to the neuroanatomical observation that progressive stages in signal processing have increasingly redundant amounts of neurons that each fire increasingly rarely (Olshausen and Field, 2004). This is no longer projected to lead to signal-specific “grandmother cells” but rather to a maximally sparse and overcomplete representation of the world given metabolic constraints.

## 3. Putting it all together: the sparsity of entrainment

The sparsity argument for entrainment is then as follows: Phenomena that contain regularities are more efficiently encoded in the frequency domain. We can therefore expect that the optimal basis, such as that obtained through SVD, would be much more similar to the frequency-domain mapping of signal, by a common similarity measure such as tangent distance (Simard et al., 1998), than the spatiotemporal mapping of the signal. Finally, over time we can expect the atoms in the brain's sparse overcomplete dictionary to minimize metabolic and computational costs by reconstructing signals along bases that are closer in tangent distance to the frequency domain than the spatiotemporal.

Returning to the descriptions of entrainment in the literature, many of the characteristic behaviors found in entrainment can be accounted for with greater conceptual economy by applying sparsity-related concepts. Entrained movement is not necessarily more skillful than rhythmically independent movement, but rather entrained movement is more efficiently coded and less computationally demanding when projected onto a frequency-domain basis. Entrainments across multiple time scales (Large, 2010) can be represented sparsely when transformed, and therefore does not necessarily pose much more computational challenge than a single-scale behavior. Non-linear coupled oscillators, such as those hypothesized to underlie entrainment (Large, 2010), have been shown to be more efficiently coded and tracked in the frequency domain (Buchli et al., 2008; Orchard et al., 2013). Perceived persistence of rhythmic structures in the absence of updated information (Large and Palmer, 2002) is explained by pursuit of the sparsest basis for the signal. Similarly, the error minimization driving predictive coding (Vuust et al., 2009) is accounted for by the least-squares optimization properties inherent in SVD diagonalization. Finally, the long tradition of fascinating studies showing that humans, while in communication with each other, synchronize from head to toe (Condon and Ogston, 1966; Trevarthen, 1979; Bernieri et al., 1988; Couper-Kuhlen, 1993; Shockley et al., 2003) is not necessarily describing a behavior of great sophistication as much as a process of economy: whatever information is being communicated between subjects is mapped internally, for each participant, onto a mathematical basis that has transformed space and time into multi-scale frequency. Entraining together allows this communication to take a more efficient form than when the subjects retain rhythmic independence. Entrainment is not virtuosity, it is sparsity.

## 4. Validating the sparsity model

What experiment might validate the hypothesis that entrainment facilitates sparse coding? While we cannot observe information coding directly, we can observe behavior, and while we do not have access to the atomic dictionaries within a subject, we can determine the SVD of a subject's actions. The singular values of the SVD further provide an effective measurement tool for how sparsely the information is encoded known as *singular value entropy* (SVE). If most of the information is sparsely packed into a small number of basis vectors, the entropy of the singular value set will be low, as some vectors will have very high singular values and most will be very low. On the other hand, if the information is encoded less sparsely, the information will be spread diffusely among the basis vectors, increasing the entropy. If entrainment aids the neurally coded JSR of a movement, than the distribution of values within the SVD of the behavior is likely to shift. In particular, entropy of the singular values can be expected to decrease, with increasing dominance of the loadings of the first singular values. If SVE of the kinematic vectors of a behavior decreases while entrained, it may be taken as evidence for a cognitive re-mapping of the action.

From this hypothesis for the cognitive impact of entrainment, a second hypothesis for entrainment-based therapy may be additionally derived: if the lasting result of a repeated entrainment-based intervention is a persistent shift in kinematic SVE of a behavior, even independent of the intervention, the SVE alteration is evidence of entrainment-driven neuroplastic change.

## 5. Conclusion

As the presence of the cochlea has long suggested to anatomists, and as neural coding theory now asserts, the brain is much more aligned to the frequency domain than our everyday, spatio-temporal accounts of the world might lead us to think. Consequently, the impact of entrainment-based instruction and therapy is likely much greater than that which can be forecasted by spatiotemporal analysis of actions. Entrainment is everywhere; entrainment is powerful; but perhaps most importantly, entrainment is sparse. A sparsity model of entrainment therapy suggests that entrainment therapy is much more than a way to scaffold the re-learning of movements: it is potentially one of the most powerful approaches to the changing of behavior in the contemporary repertoire.

### Conflict of interest statement

The author declares that the research was conducted in the absence of any commercial or financial relationships that could be construed as a potential conflict of interest.
